# Prospects and Limitations of Using Endogenous Neural Stem Cells for Brain Regeneration

**DOI:** 10.3390/genes2010107

**Published:** 2011-01-14

**Authors:** Naoko Kaneko, Eisuke Kako, Kazunobu Sawamoto

**Affiliations:** Department of Developmental and Regenerative Biology, Institute of Molecular Medicine, Nagoya City University Graduate School of Medical Sciences, Nagoya, Aichi 467-8601, Japan; E-Mails: naokoka@med.nagoya-cu.ac.jp (N.K.); ekako@med.nagoya-cu.ac.jp (E.K.)

**Keywords:** subventricular zone, neuronal migration, regenerative medicine, neuronal regeneration, remyelination

## Abstract

Neural stem cells (NSCs) are capable of producing a variety of neural cell types, and are indispensable for the development of the mammalian brain. NSCs can be induced *in vitro* from pluripotent stem cells, including embryonic stem cells and induced-pluripotent stem cells. Although the transplantation of these exogenous NSCs is a potential strategy for improving presently untreatable neurological conditions, there are several obstacles to its implementation, including tumorigenic, immunological, and ethical problems. Recent studies have revealed that NSCs also reside in the adult brain. The endogenous NSCs are activated in response to disease or trauma, and produce new neurons and glia, suggesting they have the potential to regenerate damaged brain tissue while avoiding the above-mentioned problems. Here we present an overview of the possibility and limitations of using endogenous NSCs in regenerative medicine.

## 1. Introduction

**Figure 1:**
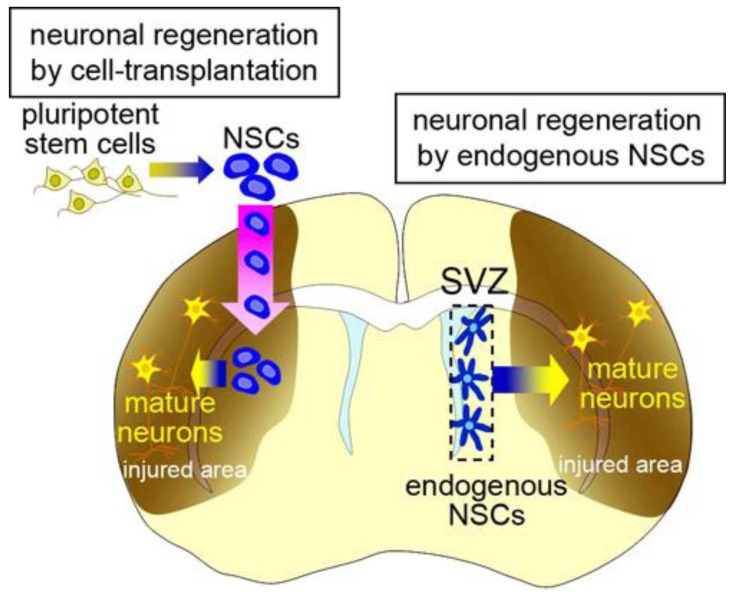
Therapeutic strategies using exogenous and endogenous neural stem cells (NSCs). Schematic drawing of a model for the therapeutic use of exogenous (left) and endogenous (right) NSCs. Exogenous NSCs derived from pluripotent stem cells including embryonic stem cells (ESCs) and induced-pluripotent stem cells (iPSCs) are transplanted into the damaged brain, and differentiate into mature neurons to replace damaged ones. Endogenous NSCs that reside in the subventricular zone of the adult brain continuously generate neurons, which migrate into the damaged area, where they replace damaged neurons.

In mammalian brain development, neural stem cells (NSCs) produce neural cells, including various types of neurons and glia. NSCs are defined as being multipotent with the capacity for self-renewal. With recent technological developments, NSCs can be induced *in vitro* from pluripotent stem cells, including embryonic stem cells (ESCs) and induced-pluripotent stem cells (iPSCs) [1–5]. The results of animal studies [6–11] support the possibility that the transplantation of these exogenous NSCs and their progeny will be a powerful strategy for regenerating nervous system tissues damaged by disease or trauma, for which no conventional treatment is available (Figure 1, left). Before this technology can be applied to patients, however, the following problems need to be resolved. First, allotransplantation provokes immunological responses to grafted donor cells, which need to be continuously suppressed. Second, pluripotent stem cells have the potential to generate tumors [10–12]. We previously established a method for isolating neural stem cells or their progenies labeled with cell-type-specific fluorescent reporters [13–15], which decreased the tumorigenicity of the transplanted cells in rats [16]. However, considering the long lifespan of humans compared with other animals, the tumorigenic risk from stem-cell transplantation should be carefully evaluated [17], particularly because, given the limited size of the intracranial cavity, a space-occupying tumor could be fatal. It was recently reported that neurons could be generated from fibroblasts by transdifferentiation without passing through the pluripotent state, which could be an efficient procedure for avoiding tumorigenic risk in cell-transplantation therapy [18]. Third, the transplantation procedure itself might injure the complicated neuronal circuitry, affecting neurological function. Furthermore, a fundamental ethical problem lies in the therapeutic use of ESCs, which are derived from blastocysts. Thus, there are serious problems associated with regenerative medicine using cell-transplantation therapy that need to be overcome before its clinical application.

Recent studies have revealed that NSCs also reside in the adult brain. They produce new neurons (neurogenesis) and glia (gliogenesis) throughout life in the subventricular zone (SVZ) at the lateral walls of the lateral ventricle, and in the subgranular zone (SGZ) in the dentate gyrus of the hippocampus [19–26]. The physiological significance of the endogenous NSCs and the mechanism that maintains functional NSCs in these specific regions of the adult brain are still unknown; however, they have the potential to regenerate lost neurons and glia in response to various pathological conditions [27,28] (Figure 1, right). This spontaneous regeneration is insufficient for structural or functional restoration of the injured brain. However, neuroregenerative therapy using endogenous NSCs is highly anticipated as an effective strategy for treating brain diseases, because it avoids the above-mentioned immunological and ethical problems and may reduce the risk of tumorigenesis. Here, we will present an overview of the function of endogenous NSCs in the adult brain and the possibility and limitations of using endogenous NSCs for brain repair.

## 2. Endogenous NSCs in the Adult Brain

In the adult brain, endogenous NSCs continuously generate new neurons in the SGZ of the hippocampal dentate gyrus and in the SVZ. Although there is no definitive marker protein that distinguishes adult NSCs [29,30], a significant portion of the NSCs express glial fibrillary acidic protein (GFAP), a marker for mature astrocytes. These NSCs have the morphological and electrophysiological characteristics of astrocytes, but they proliferate continuously and generate new granule cells in the dentate gyrus and interneurons in the olfactory bulb. In spite of their multipotency in early postnatal life or when cultured under specific conditions, adult NSCs mostly generate neurons under physiological conditions.

### 2.1. The NSCs in the SGZ

**Figure 2:**
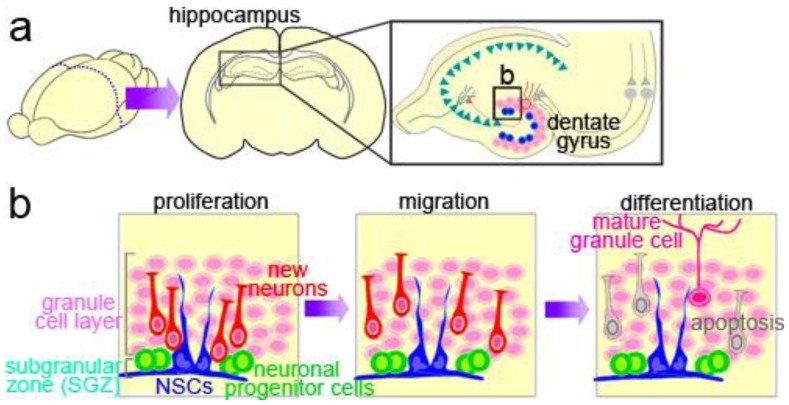
NSCs in the hippocampus. (**a**) Location, structure, and neuronal circuitry of the dentate gyrus in the hippocampus of the adult rodent brain. The input to the hippocampus is mainly provided by the entorhinal cortex through the perforant path (gray) to the granule cells (pink) in the dentate gyrus; (**b**) Neurogenesis in the dentate gyrus. NSCs (blue) and neuronal progenitor cells (light green) reside in the SGZ, where they proliferate, and generate immature new neurons (red) (left). The new neurons migrate into the granule cell layer (middle), where some of them differentiate into mature granule cells (pink), and the rest are eliminated by apoptotic cell death (gray) (right).

The hippocampus is part of the limbic system, which has important functions in learning and memory and in regulating emotion and mood. Neuronal input from the neocortex to the hippocampal circuitry passes through the dentate gyrus, which is largely composed of neurons called granule cells inhabiting the granule cell layer (GCL) (Figure 2a). NSCs, referred to as type-1 cells, reside in the SGZ, a thin cell layer between the GCL and the dentate hilus, and slowly proliferate to generate intermediate neuronal progenitors, type-2 and type-3 cells [31]; these cells produce new neurons [32–34] (Figure 2b). After a short-distance migration into the granule cell layer overlying the SGZ, the new neurons finally differentiate into mature granule cells, which are glutamatergic neurons, and are integrated into the neural circuitry [35].

A large number of the new neurons die before functional maturation, and only some of them are stably integrated into the neural network [36,37]. A recent study using a tamoxifen-induced recombination system to activate the expression of a reporter gene permanently in the progenies of NSCs showed that adult neurogenesis makes a relatively minor contribution to the neuronal population of the dentate gyrus [38]. However, notably, the newly generated immature neurons show unique electrophysiological activities, distinguishable from those of mature granule cells [39]. Their distinct electrophysiological characteristics may indicate that the new neurons play an important role in the hippocampal circuitry, despite their small numbers.

A number of studies have demonstrated that new neurons are involved in learning and memory [40–42]. Several hippocampus-dependent learning tasks increase the proliferation of neuronal progenitors in the SGZ and/or promote the survival of new neurons, and the performance of these tasks by animals correlates positively with the amount of new-neuron generation [40,43]. Moreover, suppression of the proliferation of NSCs and neuronal progenitor cells by irradiation or anti-mitotic drug treatment impairs the animals' performance [42,44].

In addition, a relationship between psychiatric symptoms and decreased hippocampal neurogenesis has been demonstrated in studies with rodents and primates [45,46]. Conversely, the chronic administration of therapeutic drugs used to treat mood disorders and anxiety disorders, including tricyclic antidepressants, serotonin-selective reuptake inhibitors, and mood stabilizers, increases neurogenesis [47,48]. The disruption of neurogenesis completely abolishes the behavioral effects of these drugs [49], indicating that the promotion of neurogenesis might be a common mechanism of action for these drugs. However, because it is difficult to establish good animal models for psychiatric diseases, and clinical studies have methodological limitations, it has not yet been possible to show directly that NSC function and hippocampal neurogenesis, or its suppression, are involved in the neuropathophysiology of these psychiatric diseases.

### 2.2. The NSCs in the SVZ

**Figure 3:**
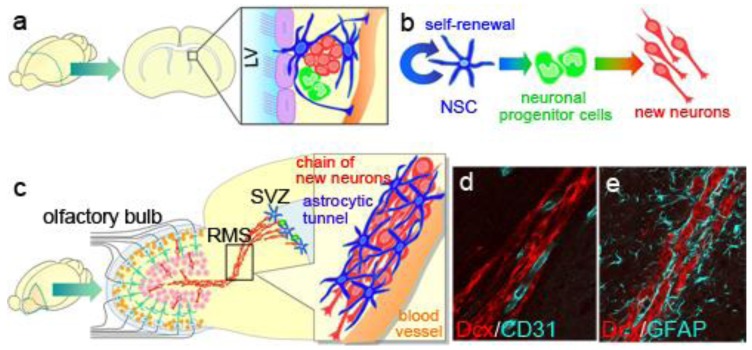
NSCs in the SVZ. (**a**) Location and structure of the SVZ. The SVZ is located at the lateral wall of the lateral ventricle and consists of four types of cells: ependymal cells (purple), which have multiple motile cilia that lie over the surface of the SVZ; multipotent and self-renewable NSCs, which have an astrocytic morphology (blue); neuronal progenitor cells (light green); and migratory new neurons (red). NSCs have an apical membrane that makes direct contact with the ventricle, and extends its processes onto the blood vessels (orange); (**b**) Generation of new neurons in the SVZ. NSCs (blue) slowly and continuously proliferate to generate new NSCs (self-renewal) and neuronal progenitor cells called “transit-amplifying cells” (light green). The transit amplifying cells proliferate quickly and produce immature new neurons (red); (**c**) Rapid and long-range migration of new neurons. Newly generated new neurons (red) in the rodent SVZ migrate rapidly and reach the olfactory bulb within a week, where they differentiate into mature interneurons. In the migratory path, called the rostral migratory stream (RMS), the new neurons form an elongated chain-like cluster, and move inside a tunnel formed by astrocytic processes (blue), which sometimes occur along a blood vessel (orange); (**d**) Chains of new neurons along blood vessels in the RMS. Sagittal brain sections were immunostained with the new neuron marker Dcx (red) and endothelial marker CD31 (light blue). Some of the chains of new neurons were extended along and closely associated with blood vessels; (**e**) Chains of new neurons surrounded by astrocytic processes in the RMS. Sagittal brain sections were immunostained with Dcx (red) and the astrocytic marker GFAP (light blue). GFAP+ astrocytic processes tightly enclosed the chains of new neurons.

The SVZ is a thin cell layer located in the lateral walls of lateral ventricles (Figure 3a). The NSCs in the SVZ are identified as a subpopulation of astrocytes [33,50] derived from radial glia, the embryonic NSCs of the ventricular zone [51]. Although adult NSCs are displaced from the ventricle by a line of ependymal cells, their apical membrane is in direct contact with the ventricle [52], which may have a role in maintaining or regulating NSC function. These NSCs also extend a long basal process that ends on blood vessels within the ventricular wall [53]. The SVZ is thought to provide a specific microenvironment, the so-called, “stem cell niche”, which enables the NSCs to maintain their self-renewing, multipotent state in the adult brain. Various proteins, including neurotrophic factors and paracrine signaling molecules, are reported to be involved in forming the niche. For example, basic fibroblast growth factor (FGF2), hepatocyte growth factor (HGF), Notch1, sonic hedgehog (SHH), Noggin, ciliary neurotrophic factor (CNTF), and a soluble carbohydrate-binding protein, Galectin-1, play important roles in stem-cell maintenance and/or self-renewal [54–59]. NSCs' sustained proliferative capacity and sensitivity to proliferative stimuli have been proposed to be involved in tumorigenic transformation [60,61], although this idea is still controversial [62].

NSCs proliferate slowly and continuously, and they generate actively proliferating intermediate progenitors called “transit-amplifying cells”, which are committed to the neuronal linage (Figure 3b). The transit-amplifying cells proliferate quickly, and their progeny become immature new neurons. We identified the Wnt-β-Catenin signal as a regulator of the proliferation and differentiation of the transit-amplifying cells that increases the pool of these cells [63]. Therefore, the proliferation of SVZ cells is controlled by a cell-type-dependent mechanism.

### 2.3. Migration of New Neurons from the SVZ

Immature new neurons generated in the SVZ have a remarkable migration activity: They migrate to the olfactory bulb at the anterior tip of the telencephalon within a week, along a pathway called the rostral migratory stream (RMS) (Figure 3c–e). Recent imaging studies successfully showed the migration of micron-sized particles of iron-oxide-labeled new neurons in living animals [64,65]. Several types of factors that regulate embryonic neuronal migration are also involved in the migration of new neurons in the adult RMS. However, a distinct mechanism is needed to enable the rapid and long-distance migration of new neurons through the densely packed mature tissue of the adult brain.

The migrating new neurons are typically bipolar, with extended leading and trailing processes, and they form elongated cell aggregates referred to as “chains”, within which new neurons can slide over and past one another [22,66]. Because the chain migration is a distinct characteristic that is not observed in the embryonic brain or anywhere else in the postnatal brain, it is important to elucidate its regulatory mechanisms. During migration in the chain, active cytoskeletal modification occurs in the new neurons. We found that cyclin-dependent kinase 5, which regulates the cytoskeleton in migrating cells in the embryonic brain, plays a crucial, cell-autonomous role in the chain formation of new neurons in the postnatal SVZ/RMS, and in the speed and direction of their migration [67]. Polysialic acid-neural cell adhesion molecule (PSA-NCAM) and β1-integrin expressed on the surface of new neurons [68,69], matrix metalloproteases produced by them, and extracellular matrix molecules including tenascin-C, proteoglycans, and laminins, have all been shown to be involved in the migration of new neurons in the RMS [68,70,71]. These molecules may regulate the adhesion between the new neurons to enable them slide past one another within a chain, despite their attachment to each other. Some of the chains of new neurons in the RMS were extended along and closely associated with blood vessels (Figure 3d), suggesting that the blood vessels may act as a scaffold for their migration [72].

New neurons are guided by various microenvironmental cues to undergo directional migration. We found that the rostral migration of new neurons occurs in parallel with the directional flow of cerebrospinal fluid (CSF) in the lateral ventricle [73]. This directional migration is disrupted by a genetic mutation that causes defective ependymal cilia development and thus a lack of normal CSF flow. Normal CSF flow creates a concentration gradient of diffusible proteins, including chemorepellents for new neurons that are secreted from the choroid plexus in the lateral ventricle, which help guide the rostral migration of new neurons against the concentration gradient. On the other hand, new neurons are attracted toward the olfactory bulb by factors including netrin1 [68], prokineticin2 [74], glial cell-line derived neurotrophic factor (GDNF) [75], and brain-derived neurotrophic factor (BDNF) [76].

**Figure 4:**
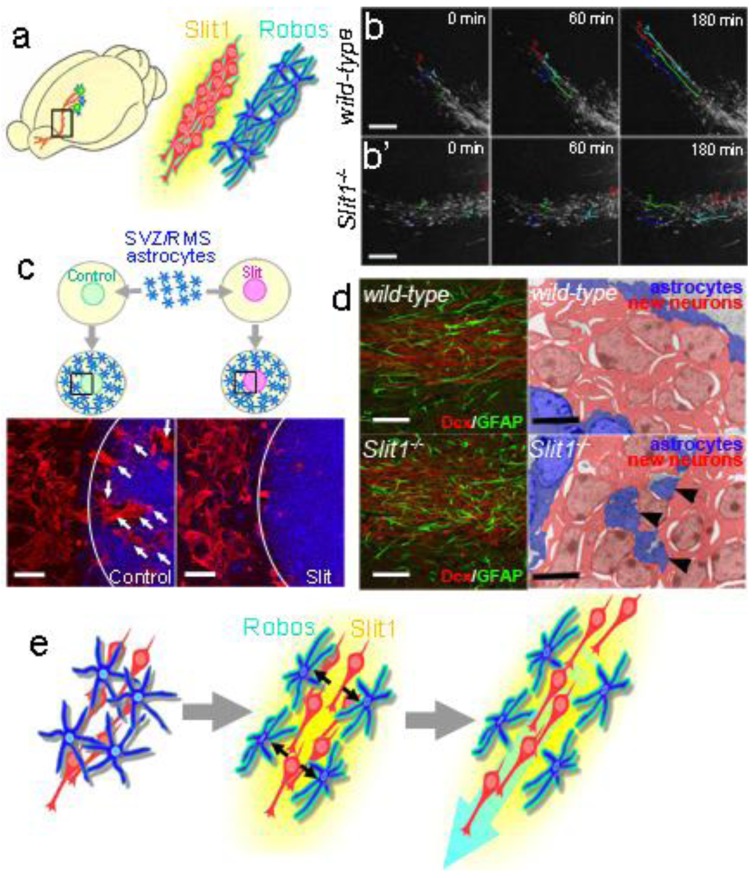
Interaction between migrating new neurons and the surrounding astrocytes. (modified from Kaneko *et al.*, Neuron, 2010 [76]). (**a**) Expression patterns of Slit1 and Robos in the RMS; (**b**) Disrupted migration of new neurons in a Slit1-defficient (*Slit1^−/−^*) brain slice. Representative paths of new neurons migrating in cultured brain slices obtained by time-lapse imaging were drawn with colored dots and lines. Compared with those of the *wild-type* (top) slice, the migration of new neurons in the *Slit1^−/−^* RMS was irregular and slower (bottom, b'). Scale bars: 200 μm; (**c**) Slit repels SVZ/RMS astrocytes. Purified astrocytes dissociated from the SVZ and RMS were co-cultured with either Slit-expressing or control HEK cells mixed into collagen gel pieces (top). After 4 days of co-culture, significantly fewer astrocytes stained with GFAP (red) were observed on the Slit-containing gel (bottom right) compared with the control gel (bottom left). Scale bars: 200 μm; (**d**) Disruption of astrocytic tunnels in the *Slit1^−/−^* RMS. Sagittal brain sections containing *wild-type* or *Slit1^−/−^* RMS were immunostained with Dcx and GFAP (left). In the wild-type RMS, GFAP+ (green) astrocytic processes were extended along the chains of new neurons (red), whereas in the *Slit1^−/−^* RMS, the arrangement of astrocytic processes was irregular. Electron micrographs (right) reveal an abnormal distribution of astrocytic processes (arrowheads) among the chain-forming new neurons in the *Slit1^−/−^* RMS. Scale bars: 50 μm (white); 5 μm (black); (**e**) Schematic drawings of the Slit-Robo-mediated interaction between new neurons and tunnel-forming astrocytes. The new neuron-secreted Slit1 and astrocyte-expressed Robo receptor control the distribution and arrangement of astrocytes to maintain the migratory path of new neurons, which assists their rapid migration through the RMS.

Notably, the chains of new neurons move inside tunnels formed by astrocytes, referred to as “glial tubes” [66,71] (Figure 3c, e). In several lines of mutant mice, aberrant astrocytic tunnel formation is accompanied by a disruption in the chain migration of new neurons [77–81], suggesting that the interaction between the new neurons and astrocytes is important for neuronal migration in the adult brain. In addition to physically separating the chains of new neurons from the surrounding tissue, which consists of a dense meshwork of neuronal fibers, astrocytes in the RMS control the migration of new neurons by taking up GABA secreted by the migrating neurons [82], trapping endothelial cell-derived BDNF [83], and secreting soluble and non-soluble factors [84,85]. We recently discovered the mechanism that forms and maintains the tunnel of RMS astrocytes: new neuron-derived soluble protein Slit1 acts on RMS astrocytes expressing Slit1's receptor Robo, which regulates the distribution and morphology of the astrocytes to form the tunnels [86] (Figure 4). Taken together, these results show that new neurons migrating in the adult RMS interact with each other and with the astrocytic tunnels, and are guided by microenvironmental cues toward the olfactory bulb.

### 2.4. Neurogenesis in the Olfactory Bulb

New neurons that reach the olfactory bulb detach from the chain, and the individual cells migrate radially into the granule cell layer (GCL) and the glomerular layer (GL), where they differentiate into olfactory interneurons, granule cells and periglomerular cells, respectively (Figure 3c). Reelin, a secreted glycoprotein, and extracellular matrix tenascin-R are involved in this process [87,88].

About a half of these new neurons are eliminated within six weeks of their birth [89], but some remain longer than a year, depending in part on the olfactory input [89,90]. The olfactory bulb is the first relay station in the olfactory system, where odor information from the olfactory epithelium is transferred to higher centers in the brain. Interneurons there modulate the activity of glutamatergic projection neurons. Although it is reported that newly added interneurons are involved in odor discrimination [91], their actual function in the olfactory circuitry remains unclear [92]. Considering that the projection neurons are never replaced, the turnover of interneurons is likely to be responsible for the plasticity of the olfactory system.

## 3. Regeneration of Neurons by Endogenous NSCs

Following the loss of neurons in various pathological conditions, including stroke, neurodegenerative diseases, and trauma, the proliferation activity of NSCs increases, and newly generated neurons appear in and around the damaged area. Recent studies on the human post-mortem brain revealed that cerebral infarction patients produce new neurons following the insult [93–95]. These findings indicate that there is a potential for neuronal regeneration in the mammalian brain, although the spontaneous regeneration is insufficient to compensate for the lost neurons, either histologically or neurologically.

**Figure 5:**
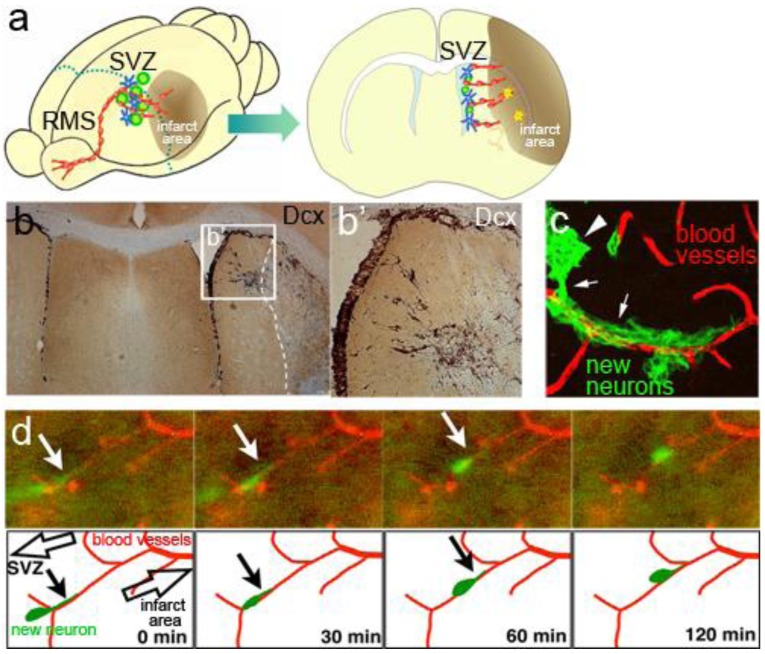
Migration of new neurons to an injured area. (**a**) Schematic drawings of SVZ new neurons migrating toward an infarcted area; (**b**) Mouse brain section 18 days after experimental ischemic stroke stained with the new neuron marker Dcx (brown). Transient middle cerebral artery occlusion (MCAO) caused infarction (white broken line). Eighteen days later, new neurons generated in the SVZ migrated toward the infarcted area. b' shows a higher-magnification image of the boxed area in b; (**c**) Association of migrating new neurons with the vasculature. A brain section 18 days after MCAO was immunostained with Dcx (green) and the endothelial marker CD31 (red). Many of the new neurons migrating toward the infarcted area were closely associated with blood vessels; (**d**) Vascular scaffold for new neurons migrating toward the infarcted area. Time-lapse imaging of a cultured brain slice after MCAO. New neurons were labeled by lentivirus injection into the lateral ventricle of Flk1-EGFP mice. A new neuron (green) extended leading process (arrows) and migrated along a blood vessel (red).

**Figure 6:**
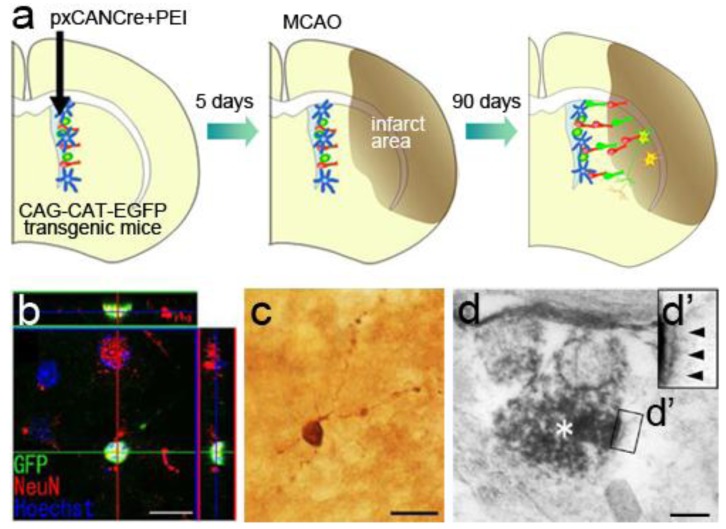
Endogenous NSC-derived neuronal regeneration. (**a**) Schematic drawing of the experimental procedure. The pxCAN*Cre* plasmid was injected into the lateral ventricle 5 days before MCAO, then the fate of GFP-labeled new neurons generated in the SVZ was detected 90 days after MCAO; (**b**) Confocal 3D reconstruction image of a GFP (green)-labeled cell expressing the mature neuronal marker, NeuN (red). Ninety days after MCAO, 29% of the SVZ-derived GFP-positive cells around the infarcted area expressed NeuN, a specific marker for mature neurons. Scale bar: 20 μm; (**c**) A GFP-positive cell exhibiting a neuronal morphology. Scale bar: 20 μm; (**d**) An electron micrograph showing a GFP-positive axon (asterisk) containing presynaptic vesicles. A higher-magnification view of the region of the boxed area (d') shows the postsynaptic density (arrowheads). Scale bar: 0.5 μm.

One of the pioneer studies of insult-induced neurogenesis showed that transient global ischemia causing the death of pyramidal neurons in the CA1 region in the hippocampus of the adult gerbil activates the proliferation of NSCs in the SGZ, increasing the number of new granule neurons in the GCL; however, the lost CA1 neurons are never replaced [96]. Another study using an adult rat model of transient global ischemia showed that NSCs/progenitors in the caudal extension of the SVZ close to the hippocampus migrate and regenerate CA1 pyramidal neurons there [97]. In addition, after focal ischemia induced by middle cerebral artery occlusion (MCAO), the most common model for ischemic stroke that causes infarction of the lateral striatum and adjacent neocortex, a small number of striatal projection neurons are regenerated [98]. That study showed that, within a week after the lesion, NSCs and progenitor cells in the SVZ begin to proliferate, and new neurons with a migratory morphology and newly generated mature neurons appear at the boundary of the damaged area in the striatum (Figure 5), but the origin of these cells was uncertain. Using viral infection-mediated cell-specific introduction of GFAP expression, we showed that these neurons are generated by GFAP-expressing NSCs in the SVZ and migrate radially into the damaged striatum, where they differentiate into mature neurons [99] (Figure 6). Thus, NSCs in the SVZ provide new neurons with a remarkable migration capacity, which may compensate for neurons lost to insult, and help regenerate the neuronal circuitry. These findings further imply that the SVZ could be an important therapeutic target for various pathological conditions.

Insult-induced alterations in the microenvironment play an important role in NSC activation. Among neurodegenerative conditions, ischemic stroke causes especially drastic biological responses soon after the lesion, due to its sudden onset. First, immune responses, including the activation of microglia and astrocytes around the infarcted area and T-lymphocyte infiltration into the damaged brain, begin [100–102]. These cells produce cytokines and other molecules that promote or inhibit the neurogenic function of the NSCs [103]. At the same time, the expression of angiogenesis-related genes, including vascular endothelial growth factor (VEGF), FGF2, and epidermal growth factor (EGF), is markedly increased in the damaged region [104]. These factors are also known to stimulate the proliferation of NSCs/progenitor cells in the SVZ [59,105,106]. Angiogenesis in the ischemic region precedes neurogenesis, and vascular endothelial cells release soluble factors that promote the self-renewal of neural stem cells in the SVZ. Notably, recent studies have shown that the vasculature in the SVZ is an important component of stem-cell niches [53,107,108], suggesting that angiogenesis plays a critical role in activating NSCs after stroke.

New neurons generated by activated NSCs in the SVZ migrate in the striatum toward the infarct area, frequently forming chain-like structures similar to those observed in the RMS. We found that these aligned cells are closely associated with astrocytic processes and blood vessels [99,109] (Figure 5c, d). Migration of these new neurons is controlled by stroma cell-derived factor 1 (SDF1) and angiopoietin 1 (Ang1), which are produced by vascular endothelial cells and by monocyte chemoattractant protein 1 (MCP1), which is expressed by activated microglia and astrocytes in the damaged area [109–113]. The signals of these molecules are mediated by their respective receptors, CXCR4, Tie2, and CCR2, which are expressed on migrating new neurons. Therefore, the migration of new neurons in the injured brain is regulated by interaction with their surroundings, which include activated glia and vasculature.

Previous reports have described BrdU-labeled newborn cells that express markers for mature neurons within the damaged striatum as early as 30 days after the induction of ischemia. We examined the phenotype of SVZ-derived GFP-labeled cells after an extended survival period by light and electron microscopy [99]. The labeled cells were found to possess long processes, express NeuN, and form synaptic structures in the damaged striatum 90 days after ischemia induction (Figure 6). These results strongly suggest that SVZ cells have the ability to generate functional mature neurons that survive in the damaged striatum for considerable periods.

On the other hand, studies have also suggested that the capacity of endogenous NSCs to compensate for lost cells is limited. In spite of the active proliferation of NSCs after insult, they become more gliogenic than neurogenic [114]. In addition, most of the new migrating neurons in and near the injured area die before differentiating into functional neurons, possibly because of a lack of factors and stimulation to support their survival and differentiation; thus, only 0.2% of the dead neurons are replaced [98]. NSCs in the SVZ do not show a neurogenic response to infarction that is within the cortex and does not involve the striatum [115,116]. Moreover, recent studies suggest that these NSCs cannot alter the types of neurons they generate depending on the context: they produce only calretinin-expressing interneurons, a subtype of interneuron that is continuously replaced in the olfactory bulb under physiological conditions, and they do not produce striatal neurons [117,118].

A similar limitation was reported in an animal model of Parkinson's disease: After the specific elimination of dopaminergic neurons by injecting 6-OHDA, the proliferation of NSCs in the SVZ was dramatically increased by treatment with transforming growth factor α, but these cells never differentiated into the neuronal lineage [119].

However, in spite of these apparent limitations to the regeneration of damaged brain tissue by endogenous NSCs, accumulating studies show beneficial effects of interventions that promote neurogenesis, including treatment with erythropoietin [120], statins [121], activated protein C [122], HDAC inhibitors [123], and EGF/FGF-2 [124], on their functional recovery following a lesion. It has not been determined whether these effects depend directly on the promotion of neuronal regeneration by NSCs, or whether accompanying events, such as enhanced glial regeneration and other types of trophic support, are more important. Moreover, a key issue in the field of neuronal regeneration is that newly generated neurons need to make the appropriate connections, although the details of this process are still largely unknown. Further studies are needed to clarify how newly generated neurons are associated with neurological improvement and to elucidate the comprehensive mechanism regulating the endogenous regeneration system.

## 4. Regeneration of Myelin by Endogenous NSCs

In the central nervous system, oligodendrocytes form the myelin sheath, an important structure for nerve conduction that wraps around axons to facilitate the rapid, saltatory conduction of electrical impulses. Demyelination is observed in both oligodendrocyte-specific degeneration diseases, such as multiple sclerosis (MS), and non-specific insults, including severe ischemia. Demyelination causes conduction block, leading to a variety of neurological impairments. Compared with neurons, oligodendrocytes are intensively regenerated by an endogenous pool of NG2 chondroitin sulfate-expressing oligodendrocyte progenitor cells, which are broadly distributed in the adult brain [125,126]. Moreover, recent studies suggest that NSCs in the SVZ are also involved in this process [127–129]. Thus, NSCs in the SVZ are considered a potential target for regenerative strategies to treat demyelination-associated pathophysiologies.

Even under physiological conditions, a small subpopulation of NSCs and progenitors in the SVZ express the oligodendrocyte lineage transcription factor, Olig2, generate oligodendrocyte progenitors. These cells express PSA-NCAM but not the neuronal lineage marker, beta3 tubulin, and migrate into the corpus callosum, striatum, and fimbria fornix, where they differentiate into nonmyelinating progenitors and mature myelinating oligodendrocytes [127,128]. Chemically induced demyelination in rodents markedly promotes this process [127,129]. Mitogens such as FGF-2 increase the production of oligodendrocyte progenitors and neuronal progenitors, whereas PDGF-AA and EGF stimulate NSCs to specifically produce NG2+ oligodendrocyte progenitors [130–132]. After their migration, the oligodendrocyte progenitors differentiate into mature oligodendrocytes and regenerate myelin on the affected axons. Recent studies have identified factors involved in this process, which include EGF [131,133,134], insulin-like growth factor-1 [135], Wnt-β-catenin mediator Tcf4 [136], Notch1 [137], and erythropoietin [120,138].

Notably, there are several limitations to the regeneration of myelin by endogenous cells. The maturation and myelination steps have been suggested to be particularly vulnerable; for example, remyelination is disturbed in both an animal demyelination model [120,139] and patients with chronic MS [140,141]. The spontaneous regeneration of myelin by endogenous cells is insufficient to completely restore the injury. However, efficient interventions that promote this process have yet to be established. Moreover, for hereditary dysmyelinating diseases in which myelin formation is developmentally disturbed, endogenous cell-based regeneration is not available. In animal models of such diseases, the allotransplantation of exogenous cells has resulted in successful myelination [142,143].

Myelination is a critical step for restoring neuronal function, not only for the demyelinated axons of surviving neurons but also for the new neurons regenerated after a lesion. Thus, for successful neuronal regeneration, the appropriate regeneration of oligodendrocytes is also needed.

## 5. Conclusion

NSCs in the SVZ are the main source of new neurons that migrate toward a lesion site, where they differentiate into mature neurons. Moreover, they might produce a small but significant number of oligodendrocytes, which contribute to remyelination. There are many challenges to overcome before the regeneration or repair of neuronal circuitry can be achieved. However, considering the fundamental advantages of endogenous NSCs for therapeutic use, free from immunological and ethical problems, the mechanisms of insult-induced neuronal regeneration and remyelination described here are of fundamental importance for understanding the molecular mechanisms that control endogenous NSCs and their progeny. For future clinical applications, interventions that regulate the migration, differentiation, survival, and functional maturation of newly generated cells to promote efficient regeneration, without over-activating the NSCs (a speculated tumorigenic risk), should be particularly important for developing novel and reliable neuronal self-repair strategies.
